# Profiling of Cytosolic and Peroxisomal Acetyl-CoA Metabolism in *Saccharomyces cerevisiae*

**DOI:** 10.1371/journal.pone.0042475

**Published:** 2012-08-02

**Authors:** Yun Chen, Verena Siewers, Jens Nielsen

**Affiliations:** Department of Chemical and Biological Engineering, Chalmers University of Technology, Göteborg, Sweden; Auburn University, United States of America

## Abstract

As a key intracellular metabolite, acetyl-coenzyme A (acetyl-CoA) plays a major role in various metabolic pathways that link anabolism and catabolism. In the yeast *Saccharomyces cerevisiae*, acetyl-CoA involving metabolism is compartmentalized, and may vary with the nutrient supply of a cell. Membranes separating intracellular compartments are impermeable to acetyl-CoA and no direct transport between the compartments occurs. Thus, without carnitine supply the glyoxylate shunt is the sole possible route for transferring acetyl-CoA from the cytosol or the peroxisomes into the mitochondria. Here, we investigate the physiological profiling of different deletion mutants of *ACS1*, *ACS2*, *CIT2* and *MLS1* individually or in combination under alternative carbon sources, and study how various mutations alter carbon distribution. Based on our results a detailed model of carbon distribution about cytosolic and peroxisomal acetyl-CoA metabolism in yeast is suggested. This will be useful to further develop yeast as a cell factory for the biosynthesis of acetyl-CoA-derived products.

## Introduction

Acetyl-coenzyme A (acetyl-CoA) is involved in many different metabolic pathways and plays a key role in linking anabolism and catabolism. It is a precursor metabolite for *de novo* biosynthesis of fatty acids and sterols. It is also the end product of lipid degradation by β-oxidation, and it is used for synthesis of C_4_ metabolites via the glyoxylate shunt during utilization of carbon sources such as fatty acids and C_2_ compounds, e.g. acetate and ethanol. Furthermore, it is a crucial substrate for energy production in the tricarboxylic acid (TCA) cycle. In addition to serving as an important metabolite intermediate in central carbon metabolism, acetyl-CoA is also an important building block for the biosynthesis of many other, biotechnologically relevant metabolites, such as waxes, flavonoids, certain amino acids, carotenoids, polyhydroxyalkanoates, polyketides, butanol and isoprenoids.

In *Saccharomyces cerevisiae*, acetyl-CoA metabolism takes place in at least four subcellular compartments: nucleus, mitochondria, cytosol and peroxisomes. Nuclear acetyl-CoA serves as acetyl donor for histone acetylation [1], but besides this role in chromatin regulation, acetyl-CoA is mainly important for the central carbon metabolism. Depending on the supply of substrate to the cell, various mechanisms may lead to the formation and utilization of acetyl-CoA. With sugars as carbon source, direct formation of acetyl-CoA from pyruvate catalyzed by the pyruvate dehydrogenase complex (PDH) in the mitochondria serves as fuel for the TCA cycle. On the other hand, acetyl-CoA generated via direct activation of acetate in an ATP-dependent reaction by an acetyl-CoA synthetase (ACS) in the cytosol is the only source for fatty acid and sterol biosynthesis [2]. During growth on oleate, acetyl-CoA is the end product of β-oxidation of straight chain fatty acids, which in yeast exclusively takes place in the peroxisomes [3]. When cells grow on a non-fermentable substrate such as ethanol or acetate, an additional extra-mitochondrial pool of acetyl-CoA is required for the glyoxylate shunt where it is consumed by citrate synthase and malate synthase.

Since the membranes of organelles are impermeable for acetyl-CoA [4], this molecule must either be synthesized within each subcellular compartment where it is required or imported using specific transport mechanisms. Two transport systems have been identified [4]. In the first, the carnitine/acetyl-carnitine shuttle, acetyl-CoA produced in the peroxisomes or the cytosol is converted into acetylcarnitine, which is subsequently transported into the mitochondria. However, it has been shown that *S. cerevisiae* is not capable of *de novo* synthesis of carnitine and unless carnitine is supplied with the medium this transport system is non-functional [5]. The second pathway is synthesis of C_4_ dicarboxylic acids from acetyl-CoA via the glyoxylate shunt, followed by transport of the C_4_ dicarboxylic acids to the mitochondria or the cytosol where they can serve as precursor for formation of pyruvate or phosphoenolpyruvate required for either the TCA cycle or for gluconeogenesis.

A hallmark of eukaryotic cells is the organization of various specific anabolic and catabolic pathways into subcellular compartments. Cast as a central molecule in yeast metabolism, it is not surprising that the biochemical mechanism for the generation and utilization of acetyl-CoA has been studied in detail. However, the compartmental demands for acetyl-CoA vary with the nutrient supply of a cell, and it has been difficult to ascertain the cellular location and function of certain enzymes, and further complicated by multiple isoforms of some of these enzymes related to acetyl-CoA metabolism in *S. cerevisiae*. This holds in particular for acetyl-CoA synthetase (EC 6.2.1.1), which catalyzes the ligation of acetate and CoA. Two structural genes, *ACS1* and *ACS2*, encoding this enzyme have been characterized in *S. cerevisiae* [6]. At high concentrations of glucose, *ACS1* is transcriptionally repressed, whereas a severe de-repression occurs under glucose limitation or supplementation with non-fermentable carbon sources such as ethanol or acetate. Acs1p was originally thought to be located in the mitochondria [7], [8], and possibly also in the peroxisomes [9]. Green fluorescent protein (GFP) tagging however suggested a distribution between the nucleus and the cytoplasm [10]. With respect to substrate specificity Acs1p was shown to have a 30-fold higher affinity to acetate than Acs2p supporting the idea that Acs1p is primarily responsible for acetate activation during growth on C_2_ compounds [6]. *ACS2* is constitutively expressed irrespectively of the kind of carbon source and type of metabolism (respiratory or fermentative) [2], [11]. Thus, it is essential for growth on glucose containing medium. It has also been demonstrated that *ACS2* is required for histone acetylation and the biology of the endoplasmic reticulum (ER), the Golgi apparatus and the vacuole [1]. Hence, although Acs2p was predicted to be cytosolic, it may also be present in the nucleus and possibly in the ER with respect to its function in histone acetylation and global transcriptional regulation. To allow for growth on fatty acids, ethanol or acetate, the glyoxylate shunt enzymes play an essential role because of the net synthesis of C_4_ metabolites from acetyl-CoA units, which are either the end product of fatty acid degradation or result from activation of C_2_ compounds (ethanol or acetate). Of the glyoxylate shunt enzymes, citrate synthase 2 (Cit2p) and malate synthase 1 (Mls1p) catalyze the condensation reaction of acetyl-CoA with oxaloacetate or glyoxylate, respectively. It has been difficult to establish whether all glyoxylate shunt enzymes are localized in the peroxisomes or in the cytosol. Cit2p was firstly determined as extra-mitochondrial by using immunological criteria [12]–[14]. From cellular fractionation and metabolic changes of a deletion mutant, different authors suggested that Cit2p is a cytosolic enzyme [12], [15]. However, Lewin and coworkers [16] concluded that it was peroxisomal and involved in the glyoxylate shunt by sedimentation on density gradients after cultivating the yeast cells on an oleate medium. An altering distribution has also been reported for Mls1p. Immunoelectron microscopy in combination with cell fractionation showed that it was peroxisomal when cells were grown on fatty acids, while it remained in the cytosol when yeast utilized C_2_ compounds such as ethanol or acetate [17].

Here we map the carbon distribution of acetyl-CoA metabolism in yeast under different nutrient conditions. Among a variety of *S. cerevisiae* strains, the CEN.PK strain family was selected based on the physiological and genetic properties [18]. We investigate the growth phenotypes and metabolite processing in different mutants affected in acetyl-CoA metabolism. Various location and transport possibilities are discussed under different nutrient situations, and a detailed map for acetyl-CoA metabolism in yeast is proposed.

## Materials and Methods

### Yeast strains, plasmid construction and gene deletions

*S. cerevisiae* strains and plasmids used in this study are summarized in Table 1. All experiments were performed in the background of CEN.PK 113-5D (*MAT*a *SUC2 MAL2*-*8* ^c^ *ura3*-*52*; P. Kötter, University of Frankfurt, Germany).

**Table 1:** List of strains and plasmid used in this study and their genotypes.

Strain	Genotype	Plasmid	Plasmid description	Source
CEN.PK 113-5D	*MAT*a *MAL2-8* ^c^ *SUC2 ura3-52*	None		Peter Kötter^a^
SCIYC05	*MAT*a *MAL2-8* ^c^ *SUC2 ura3-52 acs1Δ::loxP*	None		This study
SCIYC23	*MAT*a *MAL2-8* ^c^ *SUC2 ura3-52 acs2Δ::loxP-kanMX-loxP*	None		This study
SCIYC06	*MAT*a *MAL2-8* ^c^ *SUC2 ura3-52 cit2Δ::loxP*	None		This study
SCIYC07	*MAT*a *MAL2-8* ^c^ *SUC2 ura3-52 mls1Δ::loxP*	None		This study
SCIYC14	*MAT*a *MAL2-8* ^c^ *SUC2 ura3-52 acs1Δ::loxP cit2Δ::loxP*	None		This study
SCIYC15	*MAT*a *MAL2-8* ^c^ *SUC2 ura3-52 cit2Δ::loxP mls1Δ::loxP*	None		This study
SCIYC16	*MAT*a *MAL2-8* ^c^ *SUC2 ura3-52 acs1Δ::loxP mls1Δ::loxP*	None		This study
SCIYC21	*MAT*a *MAL2-8* ^c^ *SUC2 ura3-52 acs1Δ::loxP cit2Δ::loxP mls1Δ::loxP*	None		This study
SCIYC28	*MAT*a *MAL2-8* ^c^ *SUC2 mls1Δ::loxP*	pIYC15	pSP-GM1 2 µ *URA3* P*_TEF1_-MLS1*-SKLΔ	This study
SCIYC29	*MAT*a *MAL2-8* ^c^ *SUC2 acs1Δ::loxP*	pIYC15	pSP-GM1 2 µ *URA3* P*_TEF1_-MLS1*-SKLΔ	This study
SCIYC35	*MAT*a *MAL2-8* ^c^ *SUC2 mls1Δ::loxP*	pIYC16	pSP-GM1 2 µ *URA3* P*_TEF1_-MLS1*	This study
SCIYC36	*MAT*a *MAL2-8* ^c^ *SUC2 acs1Δ::loxP*	pIYC16	pSP-GM1 2 µ *URA3* P*_TEF1_-MLS1*	This study

a University of Frankfurt, Germany.

To remove the SKL codons from the *MLS1* gene, PCR was applied to the genomic DNA of CEN.PK 113-5D using oligonucleotides as follows, MLS1-SKL-forward, GCCGCG*ACTAGT*AAAACAATGGTTAAGGTCAGTTTGGA; MLS1-SKL-reverse, GCAGACT*GAGCTC* **TCA**CAAATCAGTGGGCGTCGC (introduced restriction sites are indicated in italics, and the stop codon is indicated in bold). The amplification product obtained was digested with *Spe*I and *Sac*I (Fermentas, St. Leon-Rot, Germany), and ligated to a *Spe*I- and *Sac*I-digested plasmid pSP-GM1 [19], generating plasmid pIYC15. To express a SKL-truncated Mls1p, pIYC15 was introduced into SCIYC07 (*mls1*Δ) and SCIYC05 (*acs1*Δ), resulting in strains SCIYC28 and SCIYC29, respectively. Similarly, a primer pair of MLS1-SKL-forward (same as above) and MLS1-reverse, GCAGACT*GAGCTC*TCACAATTTGCTCAAATCAGTGG, was used to amplify the intact *MLS1* gene. Then the product was cloned into vector pSP-GM1 using *Spe*I and *Sac*I, resulting in plasmid pIYC16. By transformation of pIYC16 into SCIYC07 (*mls1*Δ) and SCIYC05 (*acs1*Δ), strain SCIYC35 and SCIYC36 were obtained, respectively.

Gene deletion was performed using a cloning-free PCR-based allele replacement method [20]. For deletion of *ACS1*, the regions upstream and downstream of the *ACS1* open reading frame were individually amplified from genomic DNA of CEN.PK 113-5D by PCR, using the following oligonucleotides: ACS1-UP-forward, AGATAATGGGGCACGACCTC; ACS1-UP-reverse, GATCCCCGGGAATTGCCATGTGTAATGATGATTTCTTTCC; ACS1-DOWN-forward, GCAGGGATGCGGCCGCTGACGCTTAGAATAGCCGCCCAGT; ACS1-DOWN-reverse, TTGCGAAGGTGTTTAGGAAG. The *kanMX* expression module was amplified in two overlapping parts from the plasmid pUG6 [21], using the following oligonucleotides: KanMX-UP-forward, CATGGCAATTCCCGGGGATCAAGCTTCGTACGCTGCAGGTCG; KanMX-UP-reverse, CCATGAGTGACGACTGAATCCGG; KanMX-DOWN-forward, GCAAAGGTAGCGTTGCCAATG; KanMX-DOWN-reverse, GTCAGCGGCCGCATCCCTGCCGACTCACTATAGGGAGACCG.

Fragments ACS1-UP/KanMX-UP and KanMX-DOWN/ACS1-DOWN, respectively, were fused to each other via PCR. The two fusion fragments were integrated into the genome by homologous recombination yielding strain SCIYC02.

For the loop out of the KanMX expression module, procedures were followed as described previously [21]. Subsequently, correct transformants were re-streaked on synthetic complete medium containing 50 mg L^−1^ uracil and 750 mg L^−1^ 5-fluoroorotic acid (5-FOA) for the loss of the plasmid pSH47 containing the *URA3* marker and the reestablishment of uracil auxotrophy. Thus, strain SCIYC05 (*acs1*Δ) was obtained.

The same approach was used for all gene deletions in this study. All oligonucleotides used to perform gene deletions can be found in Table S1.

The deletion of each gene was verified by diagnostic PCR, which was performed by using the genomic DNA from each strain as template, and for each gene two pairs of primers were used, one outside of the integration locus and one inside the integrated fragment (see Table S2 for the primers used for strain confirmation).

### Media and growth conditions

*E. coli* cells were grown in LB (Luria-Bertani broth) medium with ampicillin (100 mg L^−1^) at 37°C. Minimal media contained 0.67% yeast nitrogen base without amino acids (Difco Laboratories, Detroit, MI, USA), 2% agar, with 2% glucose, 2% glycerol, 2% ethanol or 0.1 M potassium acetate at pH 6.0 as carbon source. Uracil (20 µg ml^−1^) was added as required.

For selection of G418-resistant transformants, YPD plates containing 1% yeast extract, 2% Bacto-peptone and 2% glucose, were supplemented with 200 mg L^−1^ Geneticin (G418 sulfate, Gibco BRL, Germany). YPG medium containing 1% yeast extract, 2% Bacto-peptone and 2% galactose, was used for activation of Cre recombinase expression. Synthetic complete medium lacking uracil contained 0.67% yeast nitrogen base without amino acids (Difco Laboratories, Detroit, MI, USA), 2% drop-out powder without uracil (MP Biomedicals, Solon, OH, USA), 2% glucose and 2% agar.

Growth characterization in liquid cultures was performed in 250 ml baffled shake flasks containing 50 ml of minimal medium with 20 g L^−1^ glucose as carbon source, which were inoculated at OD_600_ 0.02 from pre-cultures. Pre-culture cells were grown in YPD medium and washed twice with sterile water. Cultures were incubated at 30°C in a rotary shaker at 180 rpm. Samples were taken during the process and the growth was measured by determining the optical density at 600 nm (OD_600_). The filtrate was analyzed using a Summit HPLC (Dionex, Sunnyvale, CA, USA) with an Aminex HPX-87H column (Bio-Rad, Hercules, CA, USA) to measure the residual glucose and external metabolites including glycerol, ethanol, acetate, pyruvate, glyoxylate and succinate [22].

### Spot assay

Strains were grown to late log-phase in liquid minimal medium at 30°C. The cells were harvested and washed twice with sterile water. After measuring the OD_600_ serial dilutions with final cell concentrations of 1×10^5^, 1×10^4^, 1×10^3^, 1×10^2^ cells per ml were prepared. From each dilution, 5 µl were spotted on minimal medium plates with glucose, acetate, ethanol or glycerol as carbon source. The plates were recorded photographically following 10 days incubation at 30°C.

## Results

### *ACS1* deletion mutant fails to grow on non-fermentable carbon sources while *acs2Δ* shows the opposite phenotype

**Figure 1:**
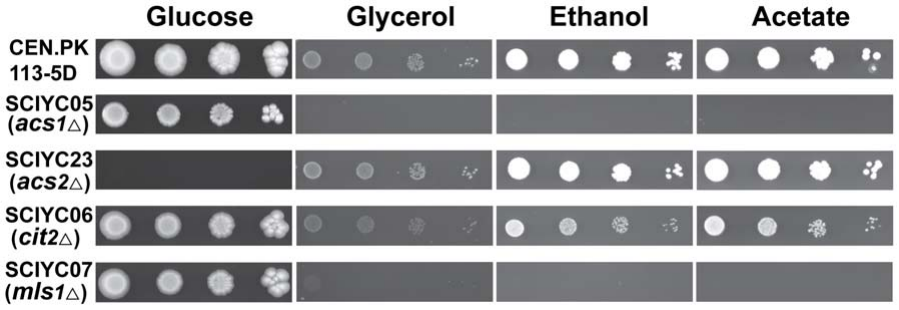
Growth phenotypes of reference and single deletion strains on minimal medium with various carbon sources. The plates were incubated at 30°C and recorded photographically 10 days after inoculation. The strains used were reference strain CEN.PK 113-5D, mutant strains SCIYC05 (*acs1*Δ), SCIYC23 (*acs2*Δ), SCIYC06 (*cit2*Δ) and SCIYC07 (*mls1*Δ).

Although two acetyl-CoA synthetases in *S. cerevisiae*, Acs1p and Acs2p, have been studied well for their function, biochemical properties and transcription regulation, there are conflicting reports on the growth phenotype of *ACS1* deletion mutants [9], [11] and some of media used before were not devoid of carnitine (using complex medium) [11]. Therefore, *ACS1* and *ACS2* were deleted in the strain CEN.PK 113-5D to generate two mutant strains, SCIYC05 (*acs1*Δ) and SCIYC23 (*acs2*Δ), respectively. The growth phenotypes of the mutant strains were re-characterized by a spotting assay on synthetic minimal medium with different carbon sources together with the reference strain CEN.PK 113-5D (Fig. 1). SCIYC05 (*acs1*Δ) showed reduced growth with glucose as carbon source compared with the reference strain. However, it failed to grow on acetate, ethanol, or glycerol as sole carbon source. This might be due to the fact that: (i) acetyl-CoA produced by Acs2p cannot enter the peroxisomes, and hence cannot enter the glyoxylate shunt which will lead to formation of C_4_ units and from here further to glycolytic intermediates required for cell growth; or (ii) the Acs2p activity is at an insufficient rate to provide the C_4_ units needed for gluconeogenesis and other biosynthetic pathways to allow cells to utilize C_2_ compounds as sole carbon source. In contrast, the *ACS2* deletion strain SCIYC23 (*acs2*Δ) was unable to grow on glucose media. But it grew normally on non-fermentable carbon sources (acetate, ethanol and glycerol) compared with the reference strain.

**Figure 2:**
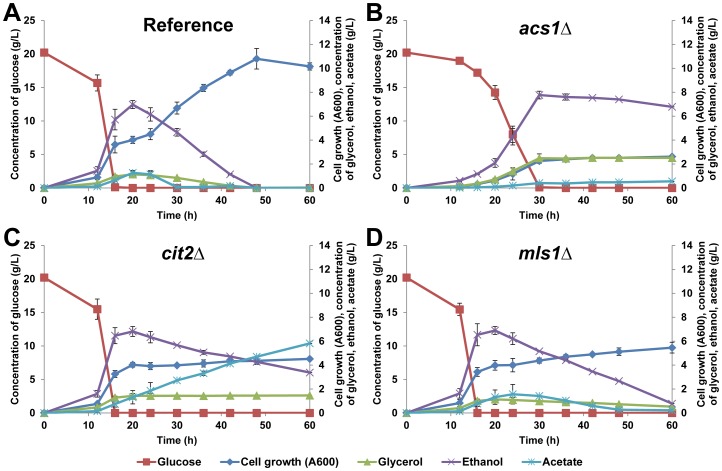
Physiological features of reference and single deletion strains. Cell growth of reference strain (A) and mutant strains SCIYC05 (*acs1*Δ) (B), SCIYC06 (*cit2*Δ) (C) and SCIYC07 (*mls1*Δ) (D), cultured in shake flask with minimal medium containing 20 g L^−1^ glucose as carbon source. All measurements are mean +/− standard error of three biological replicates.

To further examine the physiological features of the *acs1*Δ strain, liquid growth assays were conducted. SCIYC05 (*acs1*Δ) and the reference strain were cultured in shake flasks with minimal media containing 20 g L^−1^ glucose as carbon source. As shown in Fig. 2A and B, SCIYC05 (*acs1*Δ) showed an extended lag-phase, a lower growth rate, and a lower final biomass concentration during the glucose phase compared to the reference strain. The extended lag phase for an *acs1* mutant during growth on glucose has been observed in previous studies as well [11]. Although *ACS1* is reportedly subjected to glucose repression [6], the strongly delayed glucose consumption indicates further that Acs1p may have other function as well, e.g. Acs1p is probably involved in chromatin regulation since a nuclear localization has been reported as well [10]. After glucose depletion, SCIYC05 (*acs1*Δ) stopped growing, while the reference strain continued growing. HPLC analysis of metabolites revealed that SCIYC05 (*acs1*Δ) did not utilize ethanol and glycerol, which were produced during growth on glucose (Fig. 2B); whereas the reference strain could metabolize all non-fermentable carbon sources derived from overflow metabolism.

### Deletion of *CIT2* results in differences in growth on solid and liquid culture with non-fermentable carbon sources

In *S. cerevisiae*, the citrate synthase isoenzymes Cit1p and Cit3p have been shown to be exclusively located in the mitochondria, whereas it has not been conclusively shown whether Cit2p is present in the cytoplasm or in the peroxisomes. Furthermore, there are conflicting reports in the literature on the growth phenotype of *cit2*Δ mutant strains [4], [13], [15], [23]. To investigate the effect of *CIT2* disruption on cell growth and metabolism strain SCIYC06 (*cit2*Δ) was re-characterized on minimal medium with glucose, glycerol, ethanol or acetate as carbon source. As shown in Fig. 1, SCIYC06 (*cit2*Δ) did not display any severe difference in growth on glucose, but a slight growth reduction on non-fermentable carbon sources compared to the reference strain. As carnitine is not present in the media, meaning that the carnitine shuttle is not active, these results imply that *CIT2* is dispensable for growth on C_2_ carbon sources, which is in contrast to the study by Swiegers *et al.* [23].

To reinforce the observation that *CIT2* deletion still allows cell growth, SCIYC06 (*cit2*Δ) as well as the reference strain were tested in liquid culture with minimal medium including 20 g L^−1^ glucose as carbon source. SCIYC06 (*cit2*Δ) did not exhibit any growth difference when utilizing glucose as carbon source, whereas it failed to continue growing when glucose was depleted (Fig. 2C). As shown in Fig. 2C, metabolite analysis of the shake flask cultivation with SCIYC06 (*cit2*Δ) revealed that acetate was formed while ethanol was being consumed. This conversion results in net NAD(P)H production in the cells and hence allows the cells to generate ATP. The phenomenon of accumulating acetate, however, indicates that despite the presence of intact *ACS1* and *ACS2* the cells were not able to further catabolise acetyl-CoA resulting in acetate accumulation and no biomass formation.

### *MLS1* deletion affects the utilization of non-fermentable carbon sources

In yeast, two enzymes, Mls1p and Dal7p, have a 81% protein sequence identity to each other. Dal7p is thought to be involved in the degradation of allantoic acid to urea, whereas only *MLS1* is highly transcribed upon growth on non-fermentable carbon sources [24]. Hence, malate synthase Mls1p is believed to be specifically involved in the glyoxylate shunt necessary for the utilization of C_2_ carbon sources. Although Mls1p ends with a C-terminal SKL tripeptide representing a peroxisomal targeting signal PTS1, selective distribution to the peroxisomes or the cytosol depending on the carbon source has been reported [17]. As expected for the *MLS1* deletion strain, no significant changes in phenotype were observed on plate assays with glucose as carbon source (Fig. 1). In contrast, SCIYC07 (*mls1*Δ) was unable to grow on minimal media with glycerol, ethanol or acetate as sole carbon sources. Our results are consistent with previous work [17]. It was therefore confirmed that *MLS1* is indispensable for the utilization of non-fermentable carbon sources. This also applies to the other key enzyme (Icl1p) unique to the glyoxylate shunt (data not shown).

In liquid culture, while SCIYC07 (*mls1*Δ) behaved similar during glucose as carbon source; the growth of SCIYC07 (*mls1*Δ) was remarkably slow after depletion of glucose, compared with the reference strain (Fig. 2A and 2D). However, extracellular metabolite analysis revealed that ethanol was almost completely metabolized, and after glucose consumption acetate first increased slightly until 24 hours after inoculation, and then decreased slowly. The concentration of glycerol was also shown to decrease very slowly, from 1.14 g L^−1^ to 0.53 g L^−1^ after glucose depletion. Comparing the consumption of (non-fermentable) carbon sources between SCIYC06 (*cit2*Δ) (Fig. 2C) and SCIYC07 (*mls1*Δ) (Fig. 2D), it was found that SCIYC06 (*cit2*Δ) metabolized ethanol to form acetate and the biomass concentration did not increase. On the other hand SCIYC07 (*mls1*Δ) consumed both C_2_ carbon sources, but still the OD_600_ only increased by 1.46, compared to the reference strain, for which the OD_600_ increased by 6.15 (Fig. 2A). This raised the question why both the accumulated ethanol and acetate were consumed, while only a small increase in biomass was observed.

**Figure 3:**
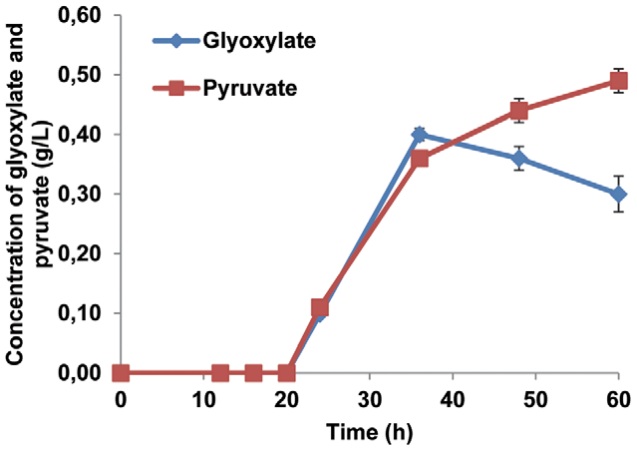
Extracellular glyoxylate and pyruvate analysis of strain SCIYC07 (*mls1*Δ). The concentrations of glyoxylate and pyruvate were measured in shake flask cultivation with minimal medium containing 20 g L^−1^ glucose as carbon source. All measurements are mean +/− standard error of three biological replicates.

Although *MLS1* was deleted, isocitrate lyase was still active in these cells producing glyoxylate, which has been reported to be a toxic intermediate for mammalian cells [25] and bacteria [26]. To test the possibility of glyoxylate accumulation, extracellular glyoxylate and pyruvate were analyzed for SCIYC07 (*mls1*Δ) as well as reference strain. No accumulation of glyoxylate or pyruvate was detected for the reference strain. For SCIYC07 (*mls1*Δ), as shown in Fig. 3, when cells started to consume both C_2_ carbon sources, glyoxylate and pyruvate accumulated. Glyoxylate reached a concentration of 0.4 g L^−1^ at 40 hours after inoculation, then decreasing a little, whilst pyruvate continuously increased from the time point of glucose depletion to the end of the cultivation.

### *CIT2* deletion restores growth in an *acs1*Δ strain

It was observed that deleting *ACS1*, *CIT2* or *MLS1*, which are related to the acetyl-CoA metabolism, led to different changes in carbon source utilization. To investigate if the double deletion or triple deletions of these three genes cause different phenotypic changes in growth characteristics compared with single deletion mutants, we constructed the mutants SCIYC14 (*acs1*Δ *cit2*Δ), SCIYC15 (*cit2*Δ *mls1*Δ) and SCIYC16 (*mls1*Δ *acs1*Δ). Repeating the *ACS1* deletion in SCIYC15 (*cit2*Δ *mls1*Δ) led to the triple deletion strain SCIYC21 (*acs1*Δ *cit2*Δ *mls1*Δ).

**Figure 4:**
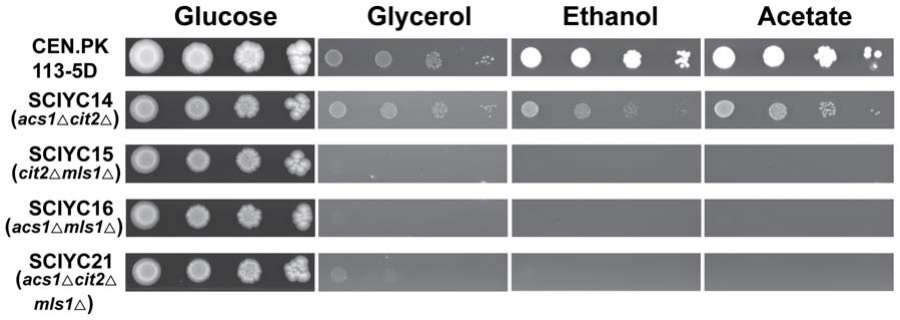
Growth phenotypes of double deletion and the triple deletion strains. Plate assay for SCIYC14 (*acs1*Δ *cit2*Δ), SCIYC15 (*cit2*Δ *mls1*Δ) and SCIYC16 (*mls1*Δ *acs1*Δ), and the triple deletion strain SCIYC21 (*acs1*Δ *cit2*Δ *mls1*Δ) on minimal medium with glucose, glycerol, ethanol or acetate as carbon source. The plates were recorded photographically after 10 days incubation at 30°C.

As shown in Fig. 4, no apparent growth was exhibited in the double deletion strains SCIYC15 (*cit2*Δ *mls1*Δ), SCIYC16 (*mls1*Δ *acs1*Δ), and the triple deletion mutant SCIYC21 (*acs1*Δ *cit2*Δ *mls1*Δ), supplemented with non-fermentable carbon sources. However, SCIYC14 (*acs1*Δ *cit2*Δ) was surprisingly able to grow on glycerol, ethanol or acetate as sole carbon source. Although the growth was slower compared to the reference strain, it clearly showed growth was restored in the *acs1*Δ and *cit2*Δ double deletion strain, whereas *acs1*Δ single deletion led to an inability to grow on such carbon sources.

### Truncated Mls1p suppresses the growth defects of the *acs1*Δ mutant

**Figure 5:**
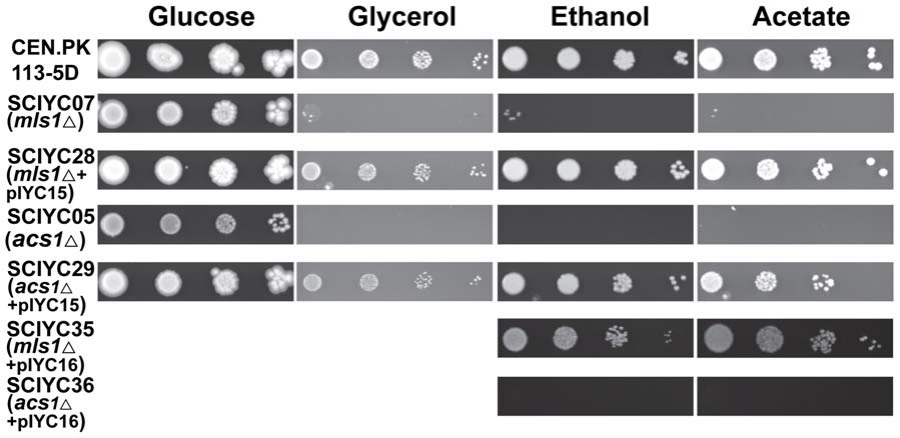
Growth phenotypes of *mls1*Δ and *acs1*Δ strains expressing an SKL-less or intact Mls1p variant. Cells were grown on minimal medium supplemented with glucose, glycerol, ethanol or acetate as carbon source. Uracil (20 µg ml^−1^) was added as required (reference, SCIYC05 (*acs1*Δ) and SCIYC07 (*mls1*Δ)). The plates were recorded photographically after 10 days of incubation at 30°C.

The inability of the *acs1*Δ single deletion strain to grow on C_2_ carbon source was overcome by further deleting another gene (*CIT2*) related to the acetyl-CoA metabolism. This phenotype implied that our previous hypothesis on the inefficiency of Acs2p in SCIYC05 (*acs1*Δ) was incorrect. This phenotypic change also indicates the possibility of flexible distribution of glyoxylate shunt enzymes. One possible assumption to explain these phenotypes would be that on non-fermentable carbon sources both Cit2p and Mls1p are localized in the peroxisomes when *ACS1* is deleted, whereas Mls1p mainly stays in the cytosol in the case of the *acs1*Δ and *cit2*Δ double deletion strain. To validate this prediction, the plasmid pIYC15 encoding a truncated Mls1p (without the -SKL tripeptide), which is supposed to be located in the cytoplasm, was transformed into SCIYC07 (*mls1*Δ) and SCIYC05 (*acs1*Δ), yielding strains SCIYC28 and SCIYC29, respectively. Harboring the plasmid pIYC15 restored the growth of SCIYC07 (*mls1*Δ) on non-fermentable carbon sources, which confirmed that expressing a cytosolic malate synthase was efficient for maintaining a functional glyoxylate shunt (Fig. 5). As shown in Fig. 5, also SCIYC29 expressing the truncated Mls1p was able to grow on glycerol, ethanol or acetate as sole carbon source. As a control, the wild type Mls1p was also expressed in SCIYC07 (*mls1*Δ) and SCIYC05 (*acs1*Δ), resulting in strains SCIYC35 and SCIYC36, respectively. While SCIYC35 grew well on ethanol or acetate, SCIYC36 was unable to grow on C_2_ carbon sources (Fig. 5). Taking into account of the growth difference of SCIYC29 and SCIYC36, the results indeed support the hypothesis that both Cit2p and Mls1p are localized in the peroxisomes during growth on non-fermentable carbon sources when *ACS1* is deleted.

## Discussion

Acetyl-CoA is a key intermediate in cellular metabolism. It is involved as a precursor metabolite; it is used for generation of Gibbs free energy, and for production of many industrially relevant compounds. In the yeast *S. cerevisiae* metabolism involving acetyl-CoA is compartmentalized and may vary with the nutrient supply of a cell. Where acetyl-CoA is metabolized on non-fermentable carbon sources is not fully understood, but as *S. cerevisiae* cannot synthesize carnitine and we did not provide this via the medium, the glyoxylate shunt is the sole possible route for transferring acetyl-CoA (in form of tricarboxylic acid cycle intermediates) into the mitochondria. Cit2p, Mls1p, Acs1p and Acs2p, are the main enzymes relevant to acetyl-CoA metabolism—either consuming or producing this molecule outside of mitochondria. Therefore, the aim of this study was to investigate the physiological effects of different mutant strains with combinations of these genes deletion (ACS1, ACS2, CIT2 and MLS1) on different carbon sources in *S. cerevisiae*, and try to understand intracellular transport of intermediates in various mutations with alternative carbon sources.

**Figure 6:**
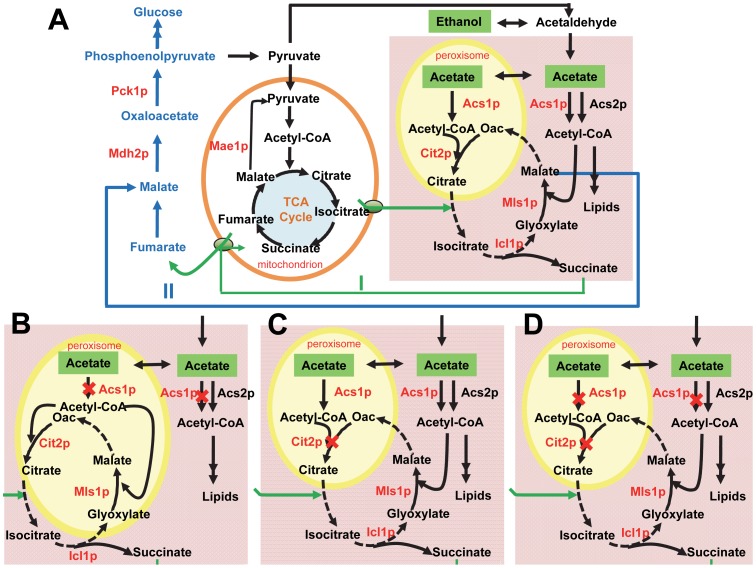
Schematic representing cytosolic and peroxisomal acetyl-CoA metabolism in *S. cerevisiae* under growth on C_2_ compounds. (A) reference strain; Acs1p is suggested to be distributed between the cytosol and the peroxisomes, Cit2p is strongly suggested to be located in the peroxisomes, Mls1p stays in the cytosol; pathway I (in green) and II (in blue) indicate the possible shunt in the absence of *CIT2* during growth on C_2_ compounds; (B) *acs1*Δ deletion strain; Cit2p and Mls1p are compartmentalized in the peroxisomes; (C) *cit2*Δ deletion strain; Mls1p remains in the cytosol; (D) *acs1*Δ and *cit2*Δ double deletion strain; Mls1p is targeted to the cytosol. Location of reactions marked by dashed lines was not identified in this study. Oac, oxaloacetate; Acs1p, acetyl-CoA synthetase 1; Acs2p acetyl-CoA synthetase 2; Cit2p citrate synthase 2; Mls1p malate synthase 1; Icl1p, isocitrate lyase 1; Mae1p, malic enzyme; Pck1p, phosphoenolpyruvate carboxykinase 1; Mdh2p, malate dehydrogenase 2.

Irrespective of the nutrient supply to the cell the cytosolic acetyl-CoA pool is indispensable for synthesis of fatty acids and sterols, and it relies completely on acetyl-CoA synthetase activity on all carbon sources. In view of its essential role in the central carbon metabolism, at least one functional isoenzyme of acetyl-CoA synthetase must be located in the cytosol. SCIYC23 (*acs2*Δ) lacking *ACS2* can grow on minimal medium containing a non-fermentable carbon source like ethanol or acetate, which clearly indicates that Acs1p is subjected to cytosolic distribution under these conditions. If Acs1p was exclusively localized in the peroxisomes, it could not activate acetate to form acetyl-CoA in the cytosol; and acetyl-CoA produced in the peroxisomes cannot be shuttled without the carnitine transport system. Our findings for the *acs2*Δ mutant are consistent with earlier reports by van den Berg and Steensma [11], i.e. that inactivation of *ACS2* did not cause inability to grow on media with acetate or ethanol as sole carbon source (in complex medium with yeast extract). Since the media used in our study are devoid of carnitine it further confirms that Acs1p is distributed to the cytosol on a non-fermentable carbon source. The ability of *acs2*Δ mutants to grow in aerobic, glucose-limited chemostat cultures has also been reported [6], which further supports the conclusion that Acs1p is subjected to cytosolic distribution under de-repressing conditions. Even though it is reported that Cit2p has both N- and C-terminal signal for mitochondrion and peroxisomes, the potential of N-terminal signal can only be recognized by removing the C-terminal sequence [27]. Taking this into account together with that recently reported *CIT2* is in peroxisomes regardless of synthetic or complex medium [28], strongly indicates that Cit2p is the peroxisomal isoenzyme of citrate synthase that is involved in the glyoxylate shunt, and it therefore seems likely that Acs1p functions in the peroxisomes in synthesis of acetyl-CoA required for use by Cit2p. Furthermore, according to the results of Kunze *et al.* [17], Mls1p remains in the cytosol in ethanol-grown cells and it therefore seems reasonable that there is a dual distribution of Acs1p for cells growing under non-repressing conditions. Similarly, it was also recently shown that fumarase and aconitase were dually localized in the mitochondria and the cytoplasm [29], [30]. Overall, Acs1p is suggested to show a dual distribution pattern between the cytoplasm and the peroxisomes, as shown in Fig. 6A.

The scenario in Fig. 6A does probably not apply to the *acs1*Δ strain. For mutant SCIYC05 (*acs1*Δ), the inability to grow on a non-fermentable carbon source was observed. Besides the requirement of 1.8 mmol cytosolic acetyl-CoA per gram cells for lipid synthesis in the cytosol [31], metabolism on C_2_ carbon sources passes through acetyl-CoA for both biosynthesis and energy metabolism. Therefore, inefficient conversion of C_2_ compounds (ethanol or acetate) to acetyl-CoA could be explained by low Acs2p activity, which may explain the *acs1*Δ strain's inability to grow on non-fermentable carbon sources. However, the double mutant strain lacking both *ACS1* and *CIT2* was shown to be able to grow on non-fermentable carbon sources, which contradicts this hypothesis. Furthermore, expressing the truncated but not the intact Mls1p in the cytoplasm of SCIYC05 (*acs1*Δ) restored the growth capacity. This observation strongly suggests that both Cit2p and Mls1p are located in the peroxisomes of cells lacking *ACS1* (Fig. 6B), and low Acs2p activity therefore cannot explain the inability of growth of the *acs1Δ* strain.

Outside the mitochondria, acetyl-CoA synthetases serve as the main producers of acetyl-CoA, while citrate synthase 2 catalyzes one of the acetyl-CoA consuming reactions. Deletion of *CIT2* breaks the integrity of the glyoxylate shunt. However, in our study the *cit2*Δ strain SCIYC06 (*cit2*Δ) was shown to be able to grow on minimal media plates containing ethanol, acetate or glycerol as sole carbon source. As shown in Fig. 6A and 5C, the disruption in the glyoxylate shunt resulting from the *CIT2* deletion can be bypassed by (i) succinate being imported into the mitochondria followed by decarboxylation of malate to pyruvate via malic enzyme (as shown pathway I in Fig. 6A); or (ii) malate being converted by cytosolic malate dehydrogenase Mdh2p to form oxaloacetate, followed by synthesis of phosphoenolpyruvate (PEP) involving PEP carboxykinase (as shown pathway II in Fig. 6A). Thus, citrate or isocitrate from the mitochondria might bypass the Cit2p reaction in the glyoxylate shunt. This is supported by the fact that the mitochondrial citrate synthase Cit1p could act as a shunt for the missing Cit2p via the citrate transporter Ctp1p [32]. Although the discrepancy of growth phenotype on solid and liquid medium is unclear so far, the growth conditions are different, which might lead to the different physiology.

Being an essential enzyme, elimination of Mls1p also breaks the integrity of the glyoxylate shunt. According to the plate assay, the *MLS1* deletion strain SCIYC07 (*mls1*Δ) was unable to grow on non-fermentable nutrient sources, indicating that Mls1p is specific and absolutely required for growth on C_2_ compounds as carbon source. Surprisingly, this mutant utilized ethanol and acetate derived from the overflow metabolism during growth on glucose in shake flask cultivation, while the biomass was increasing only slightly. After conversion of C_2_ carbon sources to acetyl-CoA, this compound can be metabolized by different pathways such as the glyoxylate shunt, or the biosynthesis of fatty acids and sterols. Analysis of the extracellular metabolite profile revealed that glyoxylate accumulated. This observation indeed showed that in absence of *MLS1*, the glyoxylate shunt was working partially, which is also consistent with the phenomenon that all metabolites from overflow metabolism are slowly consumed. These results suggested an alternative pathway to the activities of Mls1p: the counter-exchange of succinate and fumarate and subsequent intra-mitochondrial conversion of succinate to fumarate, together with the canalization of fumarate to malate. This is supported by the fact of that the mitochondrial succinate-fumarate carrier Sfc1p is indispensible for acetate utilization on minimal medium [28].

In contrast to the inability of SCIYC05 (*acs1*Δ) to grow on non-fermentable carbon sources, SCIYC14 (*acs1*Δ *cit2*Δ) was observed to be able to grow under such carbon conditions. Although the membranes of subcellular organelles are permeable to C_2_ compounds such as ethanol and acetate, metabolism on C_2_ carbon source has to pass through acetyl-CoA for biosynthesis and energy requirement. In SCIYC14 (*acs1*Δ *cit2*Δ), the conversion of a C_2_ carbon source to acetyl-CoA can be realized only by the sole extra-mitochondrial producer, cytosolic Acs2p. The acetyl-CoA units so produced cannot be transported into other compartments without the help of carnitine. Furthermore, the resulting acetyl-CoA units in the cytosol must be channeled into the synthesis of C_4_ dicarboxylic acids. Taken together with the deficiency of Cit2p activity, Mls1p which is the only candidate enzyme left can condense acetyl-CoA to form C_4_ metabolites, may well be localized in the cytoplasm of SCIYC14 (*acs1*Δ *cit2*Δ) (Fig. 6D).

In this study, we have investigated the physiological profiling of *S. cerevisiae* mutants affected in acetyl-CoA metabolism. Through analyzing the growth phenotypes and metabolite processing changes in different mutants on non-fermentable carbon sources, we have proposed a model of acetyl-CoA metabolism to speculate how various mutations alter carbon distribution. This study should be of interest to those studying acetyl-CoA production in eukaryotic cells and central carbon metabolism in fungi.

## Supporting Information

Table S1 **List of primers used in this study for gene deletions.**
(DOC)

Table S2 **List of primers used in this study for strain confirmation.**
(DOC)
