# Supplementary material for: Profiling of Cytosolic and Peroxisomal Acetyl-CoA Metabolism in Saccharomyces cerevisiae
Source: PLoS One. 2012 Aug 2;7(8):e42475. doi: 10.1371/journal.pone.0042475 (PMC3411639; doi:10.1371/journal.pone.0042475)
Supplement: Table S1 — List of primers used in this study for gene deletions. (DOC) [file pone.0042475.s001.doc]

**Table S1. List of primers used in this study for gene deletions.**

| **Primers** | **Sequence (From 5’ to 3’)** |
| --- | --- |
| ACS1-UP-F | AGATAATGGGGCACGACCTC |
| ACS1-UP-R | GATCCCCGGGAATTGCCATGTGTAATGATGATTTCTTTCC |
| ACS1-DW-F | GCAGGGATGCGGCCGCTGACGCTTAGAATAGCCGCCCAGT |
| ACS1-DW-R | TTGCGAAGGTGTTTAGGAAG |
| CIT2-UP-F | ACCGTCTTATTTACACTCCG |
| CIT2-UP-R | GATCCCCGGGAATTGCCATGTGTTGATATTGTTCCC TGAA |
| CIT2-DW-F | GCAGGGATGCGGCCGCTGACCTACTTTTACACCCCTCTGC |
| CIT2-DW-R | TGATACTAACCTGACCCCTC |
| MLS1-UP-F | ATTCCCGCAGGGTAATAAA |
| MLS1-UP-R | GATCCCCGGGAATTGCCATGGATGATAGGAGCCCGAGTC |
| MLS1-DW-F | GCAGGGATGCGGCCGCTGACTGCTTCGTTTCGTAGTTAG |
| MLS1-DW-R | CTGGTGGTCTGTGGTTGTA |
| ACS2-UP-F | TACTATTTACTTTCGTGGCAA |
| ACS2-UP-R | GATCCCCGGGAATTGCCATGGATTTACTTTCCTGTATTCTG |
| ACS2-DW-F | GCAGGGATGCGGCCGCTGACTTCCCAACTTTTGC TCTATTC |
| ACS2-DW-R | ACACCGACAGTGATGTTTCTC |
| KanMX-UP-F | CATGGCAATTCCCGGGGATCAAGCTTCGTACGCTGCAGGTCG |
| KanMX -UP-R | CCATGAGTGACGACTGAATCCGG |
| KanMX -DW-F | GCAAAGGTAGCGTTGCCAATG |
| KanMX -DW-R | GTCAGCGGCCGCATCCCTGCCGACTCACTATAGG GAGACCG |

The underlined sequences correspond to the overlapping nucleotides.
