# Supplementary material for: Profiling of Cytosolic and Peroxisomal Acetyl-CoA Metabolism in Saccharomyces cerevisiae
Source: PLoS One. 2012 Aug 2;7(8):e42475. doi: 10.1371/journal.pone.0042475 (PMC3411639; doi:10.1371/journal.pone.0042475)
Supplement: Table S2 — List of primers used in this study for strain confirmation. (DOC) [file pone.0042475.s002.doc]

**Table S2. List of primers used in this study for strain confirmation.**

| **Primers** | **Sequence (From 5’ to 3’)** |
| --- | --- |
| ACS1-UP-O | GCCACCACCATCAAGAAG |
| ACS1-UP-I | TCATTGGCAACGCTACCT |
| ACS1-DW-I | GCAGACCGATACCAGGAT |
| ACS1-DW-O | CGGAGTAGTGTCGTTTGTG |
| CIT2-UP-O | AACCAAACTACGGCATAC |
| CIT2-UP-I | CTGAGCGAGACGAAATAC |
| CIT2-DW-I | CTGCCTCGGTGAGTTTTC |
| CIT2-DW-O | AGGAGCGTTCATCTTGGT |
| MLS1-UP-O | CATCATTCAACTTTCCTCA |
| MLS1-UP-I | CTCAGTGGCAAATCCTAA |
| MLS1-DW-I | AGACTAAACTGGCTGACG |
| MLS1-DW-O | TTCTTATACATTTCCTGACTG |
| ACS2-UP-O | CTTACACCGTGAGCTTCA |
| ACS2-UP-I | CCGTCAGCCAGTTTAGTC |
| ACS2-DW-I | TACTCACCACTGCGATCC |
| ACS2-DW-O | GTACCAGACATTGACCCA |
| ACS1-F-O | AAACATGCCACCACCATC |
| ACS1-R-O | CGTCGTCGTAGCGTCAAT |
| CIT2-F-O | GAGCGAAGGCTGAAAATC |
| CIT2-R-O | ATGTGCCCGTAGGTAGAG |
| MLS1-F-O | CGTGCTTAGTGATGTCTCA |
| MLS1-R-O | AATTATCGAGGTTGTGGTAT |
